# Skin fungal community and its correlation with bacterial community of urban Chinese individuals

**DOI:** 10.1186/s40168-016-0192-z

**Published:** 2016-08-24

**Authors:** Marcus H. Y. Leung, Kelvin C. K. Chan, Patrick K. H. Lee

**Affiliations:** 1 B5423-AC1, School of Energy and Environment, City University of Hong Kong, Tat Chee Avenue, Kowloon, Hong Kong; 2 SeqMatic LLC, 44846 Osgood Road, Fremont, 94539 CA USA

**Keywords:** Skin, Mycobiome, Fungus, ITS1, Bacterial community, Microbial diversity, Pan-microbiome, Mycobiota

## Abstract

**Background:**

High-throughput sequencing has led to increased insights into the human skin microbiome. Currently, the majority of skin microbiome investigations are limited to characterizing prokaryotic communities, and our understanding of the skin fungal community (mycobiome) is limited, more so for cohorts outside of the western hemisphere. Here, the skin mycobiome across healthy Chinese individuals in Hong Kong are characterized.

**Results:**

Based on a curated fungal reference database designed for skin mycobiome analyses, previously documented common skin colonizers are also abundant and prevalent in this cohort. However, genera associated with local terrains, food, and medicine are also detected. Fungal community composition shows interpersonal (Bray-Curtis ANOSIM = 0.398) and household (Bray-Curtis ANOSIM = 0.134) clustering. Roles of gender and age on diversity analyses are test- and site-specific, and, contrary to bacteria, the effect of household on fungal community composition dissimilarity between samples is insignificant. Site-specific, cross-domain positive and negative correlations at both community and operational taxonomic unit levels may uncover potential relationships between fungi and bacteria on skin.

**Conclusions:**

The studied Chinese population presents similar major fungal skin colonizers that are also common in western populations, but local outdoor environments and lifestyles may also contribute to mycobiomes of specific cohorts. Cohabitation plays an insignificant role in shaping mycobiome differences between individuals in this cohort. Increased understanding of fungal communities of non-western cohorts will contribute to understanding the size of the global skin pan-mycobiome, which will ultimately help understand relationships between environmental exposures, microbial populations, and the health of global humans.

**Electronic supplementary material:**

The online version of this article (doi:10.1186/s40168-016-0192-z) contains supplementary material, which is available to authorized users.

## Background

The human skin is colonized by a diverse community of microorganisms consisting of bacteria, fungi, viruses, and parasites. In particular, the majority of fungi on skin are of commensal nature, but some are occasionally capable of causing a range of skin conditions [1–8]. Individuals with compromised cutaneous immunities have also been associated with altered epithelial mycobiomes, and fungal species have been shown to modulate expression of host molecules involved in changes in epithelial physiology [9]. Furthermore, fungi on skin are involved in potential associations with bacteria, modulating their physiology and virulence [10, 11]. Therefore, fungi constitute an integral member of the overall skin microbiome, and the characterization of epithelial fungal communities (mycobiomes) is essential in order to enhance our understanding on the roles of fungi in human health.

Thanks to culture-independent, high-throughput sequencing technology, our understanding of the skin microbial community has expanded in the past decade. Current skin bacterial community studies [12–20] demonstrate that microbial assemblages differ at an individual, household, and population level, and various lifestyles and environmental exposure differences have been associated with variations in the microbiome. Unfortunately, our current understanding of this enlarged microbiome is largely within the realms of the bacterial world. In contrast, there has been a paucity of knowledge regarding skin fungal communities, especially that of non-western populations. The majority of culture-independent analyses of epithelial eukaryotic communities have thus far been limited to Sanger sequencing-based studies [4, 21, 22], or involving only a small number of healthy subjects [4, 22]. At present, only a handful of large-scale mycobiome works based on high-throughput sequencing are available, most of which are based on individuals residing in the United States (USA) [10, 23–25]. Given the association between fungi and occurrences of skin conditions, understanding the baseline mycobiomes of healthy individuals undoubtedly offers valuable biological insights, ultimately increasing our understanding on how the fungal communities and human host and other microbes interact, and how different factors shape the mycobiome. Also, mycobiome analyses of non-western subjects will allow researchers to appreciate the idea of a skin “pan-mycobiome,” a global collection of mycobiome that embodies the fungal community detected on skin surfaces of host populations worldwide, similar to what is observed in bacterial communities [12, 18, 26]. However, the current lack of cutaneous fungal community data in non-western hosts impedes the determination of the skin pan-mycobiome.

Therefore, to supplement our previous investigation on the skin bacterial communities of Hong Kong (HK) individuals [12], the first large-scale high-throughput sequencing-based skin mycobiome analysis of the Chinese is described here. In this study, the mycobiome of multiple skin sites of healthy Chinese individuals residing in HK are characterized. In addition, the bacterial communities of the same individuals from our previous investigation [12] are compared with fungal data to shed light into the nature of cross-domain correlations on the skin of hosts. We also discuss how this study paves way for future mycobiome characterizations, ultimately enabling the systematic determination of a global pan-mycobiome, encompassing fungal community data across continental populations.

## Methods

### Sample collection and processing

Data included in this study originates from 40 individuals recruited as part of an investigation analyzing microbiota of residential household air, surfaces, and occupants’ skin [12, 13]. All subjects in this study are Chinese, long-term residents of HK, and not offsprings of interracial marriages. All subjects of this study were asymptomatic during sampling and have not had taken antibiotics and antifungals at least 3 months prior to sampling. The individuals in this study were living in 17 households throughout rural and urban parts of HK to cover a broad local geographical scope. Individuals and household were selected to cover a range of age and lifestyle choices such as smoking, pets, and allergies. All households involved in this study did not use pesticide or have purchased new furniture up to 3 months prior to sampling. Subjects were instructed to not wash their hands or shower prior to sampling. Metadata for samples including individual and household information is included in our previous work [12]. A total of 200 skin surface swab samples from the forehead, forearm, and palm sites were included in the work presented here, and the bacterial communities in the same samples were previously analyzed [12, 13]. In brief, autoclaved swabs were moistened with a sterile swab solution (0.15 M NaCl with 0.1 % Tween 20) [14] and each surface was sampled for 15 s covering an area of approximately 15 cm^2^, by swapping the cotton tip along the surfaces in back-and-forth motions. For forearm and palm sites, both left and right sides were sampled. Samples were subsequently stored in −80 °C within 1 h of sampling and until DNA extraction. Sterile swabs that had not been in contact with skin surfaces were included as negative swab samples. All samples, including negative control swab samples, were collected, and genomic DNA (gDNA) was extracted using the PowerSoil DNA Isolation Kit (MO BIO Laboratories, Inc., Carlsbad, CA, USA), with modification. Briefly, heat lysis was performed in 70 °C for 10 min following the addition of C1 lysis buffer, and mechanical bead beating step was performed on the Mini-Bead Beater 16 (Biospec Products, Bartlesville, OK, USA) for 10 min to enhance chemical, heat, and mechanical lysis. Blank swabs were included in the gDNA extraction step to assess for possible contamination. Purified gDNA samples were sent to SeqMatic LLC (Fremont, CA, USA) for PCR, sequence library preparation, and sequencing.

### PCR, sequence library preparation, and sequencing

PCR with primers targeting the first fungal internal transcribed spacer region (ITS1) was performed (ITS1-18S*fw*: 5′-GTA AAA GTC GTA ACA AGG TTT C-3′ and ITS1-5.8S*rv*: 5′-GTT CAA AGA YTC GAT GAT TCA C-3′) [10]. The ITS1 region was chosen over ITS2 in this study as the ITS1 region presents greater sequence diversity and resolution than the adjacent 18S and ITS2 regions [10, 27]. PCR reactions were performed in triplicate for each sample. Each 10-μL amplicon-PCR reaction consisted of 5 μL of TailorMix 2× SYBR Green qPCR Master Mix (SeqMatic, Fremont, CA, USA), 0.5 μM of each primer and 1 μL of DNA template. Each sample was denatured at 95 °C for 10 min before undergoing 35 cycles of 95 °C for 30 s, 50 °C for 30 s, and 60 °C for 1 min and a final extension at 60 °C for 10 min. The amplicons from each triplicate were pooled, purified with the DNA/RNA Purification Beads (SeqMatic, Fremont, CA, USA), and re-suspended in 60 μL of 10 mM Tris (pH 8.5). Three microliters of each purified amplicon was subjected to the indexing-PCR, with 15 μL of TailorMix 2× SYBR Green qPCR Master Mix (SeqMatic, Fremont, CA, USA), 0.5 μM of each forward (Index Primer 1) and reverse (Index Primer 2) indexing primer in a final reaction volume of 30 μL. Each sample was denatured at 95 °C for 10 min, followed by eight cycles of 95 °C for 20 s, 50 °C for 30 s, and 72 °C for 1.5 min and a final extension at 72 °C for 10 min. Indexing-PCR efficiency was monitored via real-time PCR with the TailorMix 2× SYBR Green qPCR Master Mix. Both amplicon-PCR and indexing-PCR were conducted on the 7500 Fast Real-Time PCR System (Applied Biosystems, Foster City, CA, USA). Equal volumes of each indexed amplicon were pooled and purified by gel electrophoresis. Libraries were prepared using the Illumina MiSeq Reagent Kit v2, and final library was quantified with the 2200 TapeStation (Agilent, Santa Clara, CA, USA) and sequenced with the MiSeq platform, generating 250-bp paired-end reads.

### Sequence analysis

Following sequencing, de-multiplexing and barcode removal were performed. A total of 7,226,928 reads from each of forward and reverse in .fastq format were overlapped and merged using FLASH [28], based on a maximum overlap of 250 bp. Quality filtering of paired-end reads was performed using the “fastq_filter” command in USEARCH [29], based on a maximum expected error of 1 error/read, reads trimmed to a uniform length of 275 bp and reads shorter than 275 bp removed, resulting in 6,297,874 reads passing quality control. These reads were clustered into operational taxonomic units (OTUs) based on 97 % identity using UPARSE with the “cluster_otu” command in USEARCH [30]. Reference-based chimera detection was performed using the “uchime_ref” command in USEARCH [31], with the recently released UNITE/INSDC representative sequence set (11 March 2015 version) [32] as reference. Taxonomic information was provided for each OTU with the “assign_taxonomy.py” QIIME script using default parameters. Both the commonly used UNITE fungal reference database [33] (2 March 2015 version, 55,404 sequences included) and the recently curated fungal reference database (23,456 sequences included) constructed previously by Findley et al. [10] were used to compare the coverage and accuracy of OTU taxonomic classification. Subsequent analyses involving taxonomic data were based on results derived from the curated database, as it provided a higher percentage of taxonomically assigned reads (see “Results” section). OTU lineages present in more than 5 % of the reads in negative controls were deemed possible contaminants and were removed from all samples. Following quality control and chimera and contaminant read removal, a total of 4,124,756 reads were retained for downstream community analysis. OTU, read count, and taxonomic information (based on Findley’s reference database) is provided in Additional file 1: Table S1.

For the comparison of reference databases on taxonomic coverage of other populations, data from two American studies were selected [10, 25]. Although the studies employed different primers, they all target the ITS1 region. The two studies include one that characterizes multiple body sites among ten asymptomatic adults (with unknown ethnicity) based in the Washington D.C. (Bethesda) area [10]. Only forehead, forearm, and palm sites were selected from this study for the comparative analysis. The study was selected as it is one of the few large-scale skin mycobiome works present. The other study examines the forehead mycobiomes of healthy occupants within a residence in Berkeley, California, as part of a built environment (BE) microbiome investigation [25]. Each skin sample collected in this study was an integrated sample that was pooled from each cohabiting occupant within a household. This study was selected for comparative analysis as skin samples were collected from occupants within their predominant BE habitat, mirroring that of the HK study. Following raw data acquisition, sequences followed the read quality control, OTU clustering, and taxonomic classification procedures as described above.

HK samples were normalized by random subsampling to a read depth of 1175 reads/sample using QIIME script “multiple_rarefactions.py” [34]. Fungal community richness (observed OTUs and Chao1 total OTU estimator), diversity (Shannon and Simpson), community membership (Jaccard distance, JD), and composition (Bray-Curtis dissimilarity, BCD) computations were performed using normalized data using QIIME scripts “alpha_diversity.py” and “beta_diversity.py” with default settings. Sixteen samples were removed from normalized analyses (all forearm sites of different households or individuals), as these samples had lower than 1175 reads. Good’s coverage of over 96 % for all remaining samples indicates sufficient normalized depth in capturing the sample microbial diversity. Ten rounds of random rarefactions were performed for each sample at this read depth. ANOSIM values based on BCD and JD were constructed in R package vegan (http://vegan.r-forge.r-project.org/). UniFrac distance was not used on fungal data, as the ITS1 region employed for sequencing is highly variable for informative and meaningful phylogenetic analyses [35].

### *Malassezia* species-level identification

Based on the taxonomic assignment of representative OTU sequences described above, reads belonging to *Malassezia* OTUs were included in species-level analysis. A species-level reference database, containing 90 ITS1 sequences of *Malassezia* species or strains, was constructed based on searching the NCBI database for the presence of terms “Malassezia” and “ITS1” on the sequence ID. The reference sequences were manually curated such that only sequences containing complete ITS1 sequences were retained. Representative reads from OTUs assigned as *Malassezia* were interrogated against the reference database using the “usearch_local” command in USEARCH [29] with a 97 % sequence identity threshold. Through this, the majority of OTUs initially assigned as *Malassezia* (96/142) were assigned to a species or strain found in the database. The remaining 46 OTUs were manually interrogated against the NCBI nucleotide database with BLAST (which also includes the 90 ITS1 sequences used in the first round of classification). These OTUs matched to partial ITS1 sequences that were excluded from the 90 ITS1 reference sequences. A database match with a known *Malassezia* species with ≥97 % identity to the OTU sequence over 95 % of the entire length of the query sequence is required to assign an OTU to a known species.

### Statistical analysis

The nonparametric Mann-Whitney (MW) and Kruskal-Wallis (KW) tests were employed to determine significance when comparing between two or more comparison groups, respectively. Where indicated, post hoc KW pairwise comparison tests for significance between individual groups were performed using the “kruskalmc” function in the R package pgirmess (http://cran.r-project.org/web/packages/pgirmess/index.html) following significant KW observations. For cross-domain α- and β-diversity correlations, the Spearman’s correlation and linear regression fit were computed in R (http://www.r-project.org). In order to determine the correlation between fungal and bacterial community richness, α- (observed OTUs, Chao1 total OTU estimation, and Faith’s phylogenetic diversity) diversity data was correlated between the two domains. For cross-domain β-diversity correlations, fungal BCD values between pairwise samples were correlated with both bacterial BCD and weighted UniFrac distances. Bacterial community diversity data was based on previous study [12], targeting the 16S V4 region (primers 515*fw*: 5′-GTG CCA GCM GCC GCG GTA A-3′; 806*rv*: 5′-GGA CTA CHV GGG TWT CTA AT-3′) [36, 37], selected to capture greater bacterial diversity compared to other hypervariable regions [38]. Spearman tests and linear regressions of cross-domain α- and β-diversity correlations were performed with R. Sparse correlations for compositional data (SparCC) was computed between all quality-filtered reads from bacterial and fungal data to detect co-abundance and co-exclusion correlations [39]. SparCC was chosen as it addresses the compositional bias introduced when correlating relative abundance data. SparCC analysis, network plots, and two-sided pseudo *p* values (*p* values ≤0.05 considered significant) based on 100 repetitions were computed on python scripts as described [12].

## Results

### Comparison of fungal reference databases on providing taxonomy to skin mycobiome

The UNITE database [33, 40] has thus far been the standard for taxonomic classification in mycobiome analyses across ecosystems [35, 41]. However, concerns regarding misclassification, and the assignment of sequences from different sexual forms of the same organisms into different taxa, call for modifications of the database [42–44]. A curated ITS database for skin mycobiome analysis was recently described [10], minimizing the aforementioned shortcomings of UNITE.

We assessed the two reference databases for their ability in providing taxonomic information to the fungal community in the skin mycobiome of the HK cohort. This is assessed here in terms of taxonomic coverage of each database, defined here as the ability to provide an OTU with a taxonomic classification. Perhaps due to the different number of reads in the databases, and the focus of the curated database on the human mycobiome, the curated database provides an increased percentage of taxonomically classified reads at the genus and order ranks, thereby providing higher taxonomic coverage, compared to the UNITE database (Additional file 2: Table S2). The two databases were also compared on data from mycobiome studies of two American cohorts that target the ITS1 region [10, 25], showing similar observations as the HK cohort (Additional file 2: Table S2), indicating that the superior taxonomic coverage of the curated database applies to other cohorts.

Comparison of taxonomy assigned to individual OTUs was performed (Additional file 2: Table S2) for the HK samples to assess for consistency between the two databases. The majority of the OTUs analyzed (454/711, 63.9 %) have consistent results between the two databases (this includes OTUs considered unclassified for both databases, see below). Ninety-five OTUs with unclassified genus information under the curated database had genus information under the UNITE database. On the other hand, a hundred and forty-six unclassified OTUs under the UNITE database were classified with the curated database. Three hundred and fourteen OTUs where both databases provided taxonomic information, most (298/314, 94.9 %) of which had consistent classification at the genus level. The remaining 16 OTUs with inconsistent taxonomic classification constitute 0.1 % of the reads in the entire dataset. Still, a hundred and fifty-six OTUs had no genus information regardless of the database used, indicating that neither database can completely provide taxonomic information for this cohort. Nonetheless, given the greater taxonomic coverage at the genus level, and that the curated database was previously designed and tested specifically on skin samples [10], the curated database is used for taxonomic analyses on the HK samples described below.

### Taxonomic overview of the skin mycobiome of Chinese individuals

**Fig. 1:**
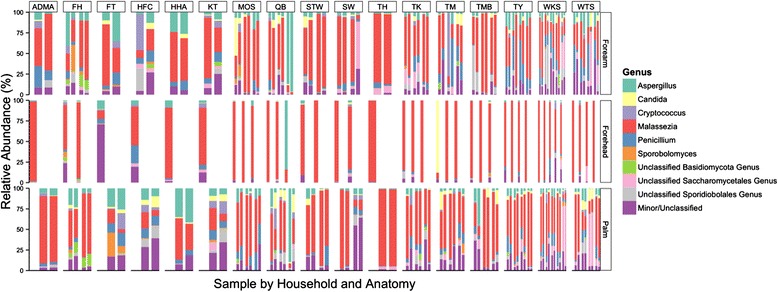
Relative abundances of fungal genera grouped by household and anatomical site. Each *bar* represents a sample for a given individual per skin site. Household locations are indicated at the *top*. For each household, forearm and palm sites have double the number of bars as forehead, as symmetrical sites were sampled. The top nine genera (including two unclassified genera of the *Basidiomycota* phylum and *Saccharomycetales* order) are represented in *different colors*, with the remaining reads classified as “Others/Unclassified”

The HK dataset contains 168 distinct and known genera. Common skin commensals including *Aspergillus* (average sample relative abundance of 8.5 %), *Penicillium* (7.6 %), *Candida* (3.1 %), and *Cryptococcus* (2.3 %) were present in all samples (Fig. 1 and Additional file 3: Table S3). A few observations could be seen when genera relative abundances were visualized across households and anatomical sites: (1) forehead sites showed the lowest diversity at this taxonomic level (79 genera detected across all forehead samples, compared to 95 and 115 genera across forearm and palm sites, respectively), mainly dominated by *Malassezia* across individuals and households; (2) for forearm and palm sites, where symmetrical samples were taken, relative abundances of major genera were generally similar between the left and right sites within most individuals (exceptions being forearm sites of individuals in HFC and FH, where larger proportions of an unclassified genus within the *Sporidiobolales* order and the *Sporobolomyces* genus were found on one side of respective individuals, and palms of individual in QB, where one side was dominated by *Aspergillus*); (3) members within a household did not necessarily share similar genera (e.g., samples between cohabitants of FH, QB, and TM).

The common skin fungus *Malassezia* constitutes an average of over 57 % of community within each sample (Additional file 3: Table S3), with a wide range of relative abundances between samples (from 2.3 to 99.7 % of reads within a sample). *Malassezia*, being a lipophilic genus, is found to be significantly more abundant in forehead samples (average relative abundance 80.9 %) than that of forearm and palm samples (50.4 and 53.6 % for forearm and palm samples, respectively, KW post hoc *p* < 0.05). *Malassezia* is also more abundant in males (average relative abundance 64.3 and 54.6 % for males and females, respectively, MW *p* < 0.01). Species-level classification of *Malassezia* reads reveals that >90 % reads belongs to *Malassezia restricta*. Nearly 2 % of *Malassezia* reads are assigned to the *Malassezia sp*. 3 and the LCP-2008a strains, potentially novel species [45]. The other common *Malassezia* colonizer, *Malassezia globosa*, is detected on 5.3 % of all *Malassezia* reads, while both *Malassezia furfur* and *Malassezia pachydermatis* are detected in low relative abundances (together <0.1 % of *Malassezia* reads). However, while non-*M. restricta* species are present in only 10 % of the entire dataset, these species are enriched in specific households and individuals (e.g., ~40 % of *Malassezia* population belongs to *M. sp.* 3 in individual QB-3Z and nearly 45 % of *Malassezia* population belongs to *M. globosa* in individual TM-3Y, Additional file 3: Table S3), highlighting the individuality of species distribution even within a genus.

Interestingly, fungi potentially associated with the local outdoor environment and local lifestyle practices are also identified. *Debaryomyces*, present in 0.3 % of the skin samples, is one of the most prevalent fungal genus in mangroves of southern China [46]. Also, genera such as *Cordyceps* and *Auricularia*, each present in 0.1 %, contains species considered to be important in traditional Chinese medicine and cuisine [47, 48]. While present in less than 0.02 % of all reads in the dataset, *Stemphylium* contains species that are pathogenic to garlic cultivation in China [49]. With the exception of *Cordyceps*, none of the genera described above are detected in the two American studies [10, 25].

### Fungal community richness, diversity, membership, and composition differ by hosts, phenotypes, and households

At a normalized sequence depth, community richness (observed number of OTUs and Chao1) and diversity (Shannon and Simpson indices) are significantly different between individuals and households (Additional file 4: Table S4). However, significance of α-diversity between other variables depends on the statistical test (Additional file 4: Table S4 and Additional file 5: Figure S1). OTU-based (i.e., observed number of OTUs and Chao1) indices reveal significant richness differences between age groups (Additional file 4: Table S4 and Additional file 5: Figure S1), driven by differences between children and adult, and children and the elderly (post hoc pairwise *p* < 0.05 for both). Foreheads (men and women combined) are significantly less diverse than other sites based on Shannon and Simpson measurements (post hoc pairwise *p* < 0.05 for both, Additional file 5: Figure S1). When all body sites are included, females confer higher diversity for Shannon and Simpson indices. When analyzed by site, gender differences are significant in foreheads and left forearms (both Shannon and Simpson diversity *p* < 0.03 for forehead and *p* < 0.006 for left forearm). α-diversity values for each sample can be found in Additional file 6: Table S5.

Analysis of similarities (ANOSIM) based on BCD (measures abundance-weighted community composition) and JD (measures presence and absence of OTUs in community, or community membership) matrices reveals significant clustering based on individual, household, and body site (Additional file 4: Table S4), with greater clustering when considering community membership for all three factors. When symmetrical sites are combined (i.e., tested for anatomy), clustering is significant only when community membership is considered, suggesting that left-right differences are mainly driven by non-abundant OTUs. Conversely, modest but significant community composition clustering by age groups is detected, suggesting that age-related changes in microbiomes are associated with changes in relative abundances of shared OTUs, similar to what is observed in bacteria [50]. Community variations between genders are insignificant by both methods.

**Fig. 2:**
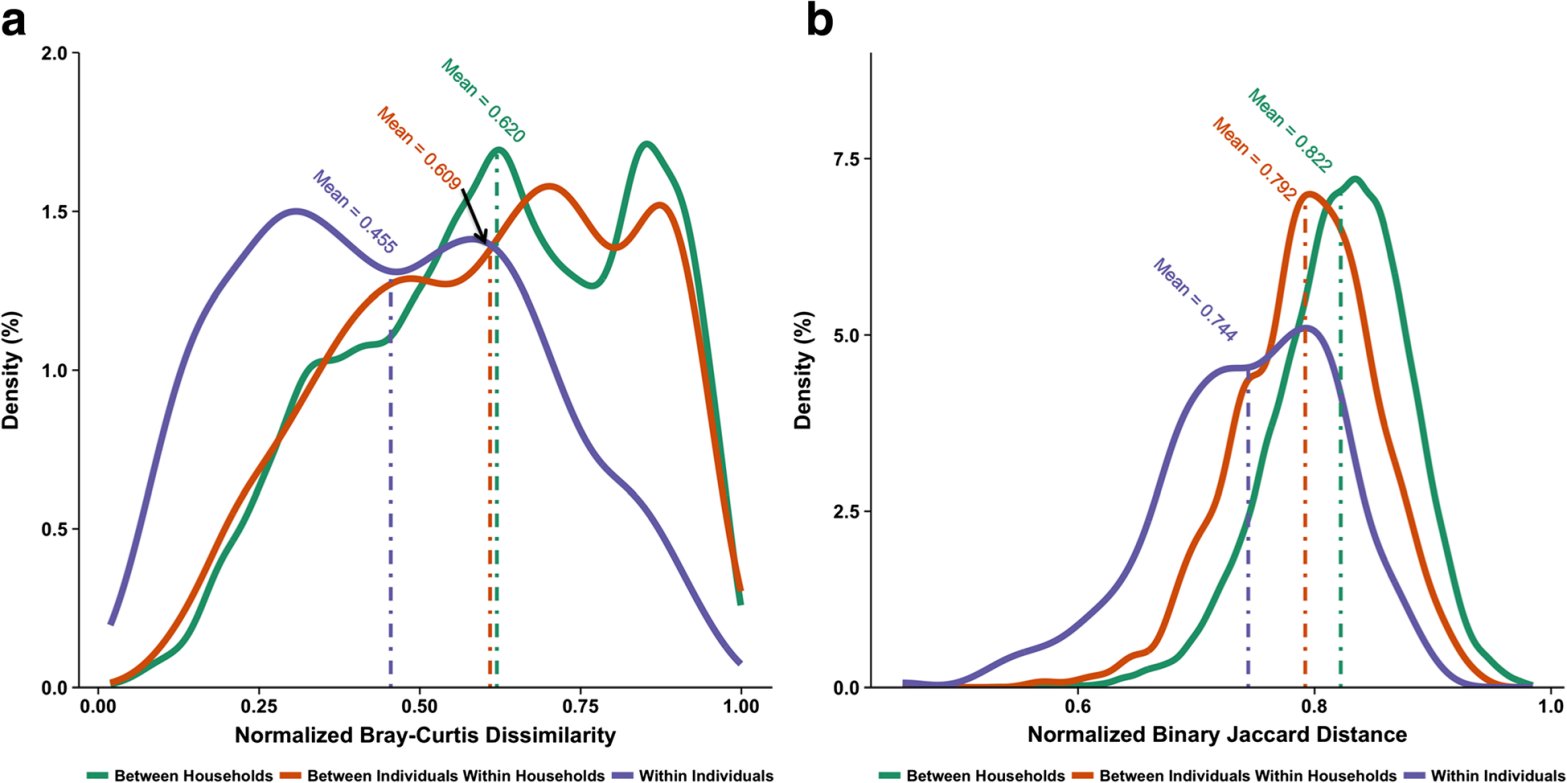
Density plots of **a** Bray-Curtis dissimilarities and **b** binary Jaccard distances between two samples depending on sample source relationships. Pairwise comparisons between samples coming from within the same individual, between different cohabiting individuals, and between non-cohabiting individuals. *Vertical line* corresponds to mean dissimilarity or distance for each of the comparison groups

To ascertain the effect of household on community differences, mean BCD and JD are compared within or between individuals (Fig. 2, Additional file 4: Table S4, Additional file 7: Figure S2, and Additional file 8: Figure S3). KW comparison reveals that the mean BCDs between samples within individuals (mean dissimilarity = 0.455) were significantly lower than that between different people (0.609 and 0.620 for within households and between households, respectively) (Fig. 2a and Additional file 4: Table S4). However, BCDs of samples between individuals are not significantly different regardless of whether these individuals are cohabitants or not (Fig. 2a and Additional file 4: Table S4). The effect of household is present only when community membership analyzed by JD is considered (Fig. 2b and Additional file 4: Table S4). When comparisons between samples are analyzed within each site (Additional file 4: Table S4 and Additional file 7: Figure S2), only the right palm shows significant community compositional differences between individuals (mean BCD of 0.608 for within-households compared to 0.660 for between households, *p* = 0.02). However, when community membership between samples within each skin site is compared (Additional file 4: Table S4 and Additional file 8: Figure S3), all sites show significant differences between within-household and between-household comparisons. Taken together, these observations are consistent with the idea that residences possibly act as an intermediate homogenizing factor when explaining the repertoire of skin mycobiome population (community membership) found on its occupants, whereas individual fungal exposure and activity differences influence community composition variations, even within a household.

In addition to individuals, dissimilarities across the entire dataset are also compared between other variables, with BCDs of the same skin sites, age groups, and gender significantly lower than that of the different groups (Additional file 4: Table S4). Interestingly, JDs showed significances that are discrepant from that of BCD analyses when looking at overall gender- and anatomy-based clustering. Within specific sites, same and different age groups showed significant compositional differences in the forehead and the left forearm. On the other hand, individual community membership clustering is significant for all separate skin sites (Additional file 4: Table S4). Overall, these observations suggest that, similar to bacteria, host and household phenotypes contribute to shaping components of skin fungal community structures. Also, we stress the importance of separating community membership and composition for community analyses, as when analyzed separately [51], they provide additional information that may be helpful in explaining complex relationships between the host and the mycobiome.

### Cross-domain comparison of fungal and bacterial communities

**Fig. 3:**
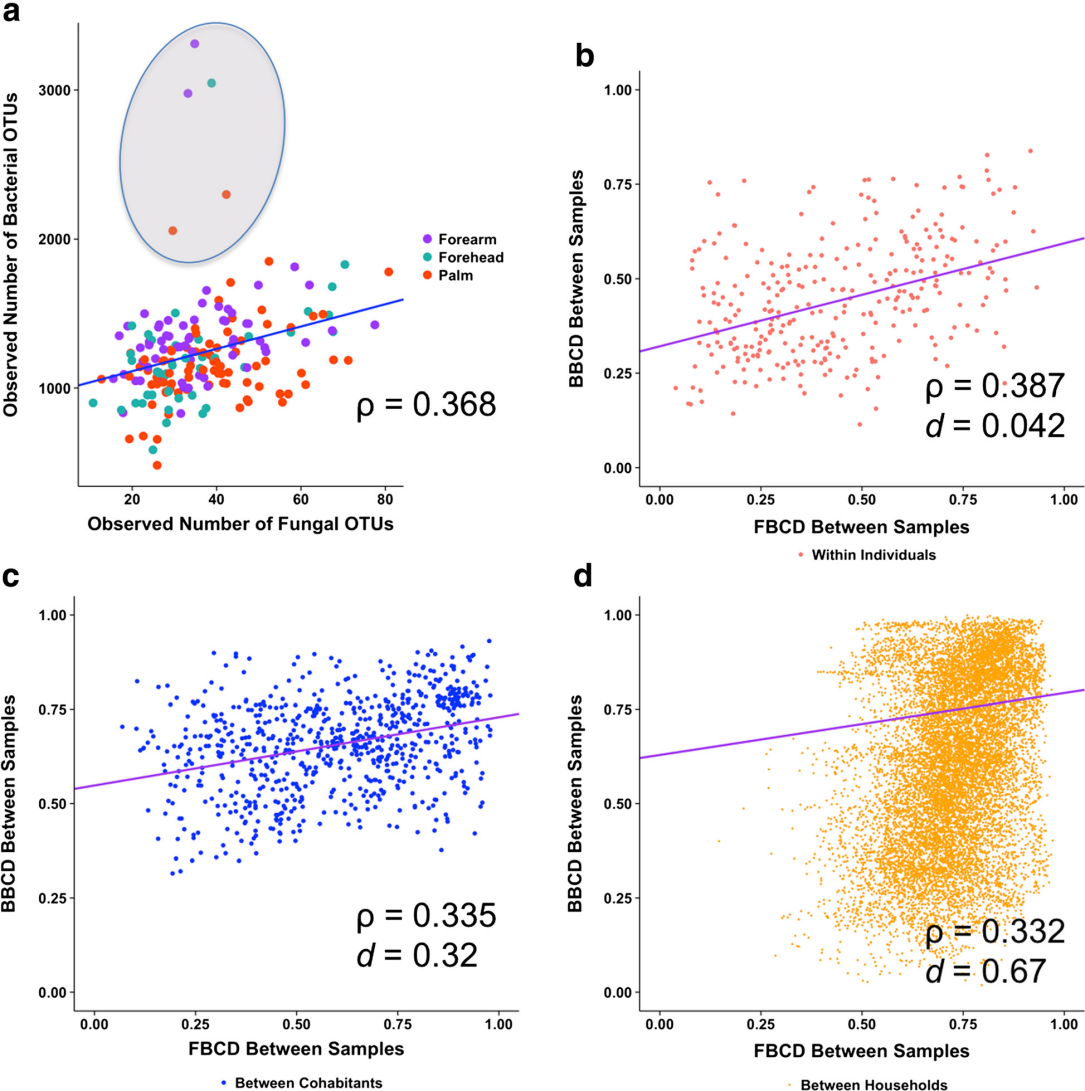
Correlation of fungal and bacterial **a** α- and **b**–**d** β-diversities. **a** Correlation of observed number of fungal OTUs and the observed number of bacterial OTUs for a given sample, colored by anatomical site. *Encircled samples* represent microbiome of one occupant in household HHA containing elevated bacterial community richness with no apparent elevated fungal richness. **b**–**d** Correlation of fungal Bray-Curtis dissimilarity (FBCD) between two samples and the bacterial Bray-Curtis dissimilarity (BBCD) of the same sample pair, grouped according to samples from **b** the same individual, **c** cohabiting individuals, or **d** non-cohabiting individuals. Spearman’s *ρ* values are provided for each plot, all with *p* < 0.0001. *Blue* and *violet lines* represent linear regression as computed in R for the respective α- (*blue*) and β- (*violet*) comparison plots

Our previous work [12, 13] analyzed bacterial communities of the same skin samples by targeting the bacterial 16S rRNA gene V4 region. Here, we combine community data from the two domains to shed light into potential cross-domain relationships between the two major branches of the skin microbiome. Respectively, rarefied bacterial [12] and fungal (from this study) richness for each sample is combined to analyze correlation between the α-diversities of the domains (Additional file 6: Table S5). Bacterial α-diversity, whether taxonomic (observed and Chao1 total estimated OTU) or phylogenetic (Faith’s phylogenetic diversity), appears to correlate positively with most of the α-diversity indices of fungal taxonomic diversity (Fig. 3a and Additional file 6: Table S5). A resident of household HHA contained a relatively elevated observed number of bacterial OTUs; however, this is not met with an elevated fungal OTU richness (Fig. 3a, encircled area), consistent with the idea that increased bacterial taxonomic diversity does not necessarily increase fungal richness, or that additional factors (such as site and occupant physiology variations) govern the relationships between bacterial and fungal community richness [10, 16]. Additional analysis involving a larger number of subjects will be required to further determine potential relationships between bacterial and fungal community richness.

To investigate correlations between bacterial and fungal community dissimilarities between any two samples, Spearman correlation was performed between fungal and bacterial BCD pairwise comparisons (Fig. 3b–d and Additional file 6: Table S5). Significantly positive cross-domain community composition correlations are identified, regardless whether the correlations between samples within an individual (Spearman’s *ρ* = 0.387, *p* < 0.001, effect size Cohen’s *d* = 0.039), between cohabitants *(ρ* = 0.335, *p* < 0.001, effect size Cohen’s *d* = 0.32), or between households (*ρ* = 0.332, *p* < 0.001, effect size Cohen’s *d* = 0.67) (Fig. 3b–d and Additional file 6: Table S5). Positive correlations between fungal and bacterial BCD are also identified for pairwise comparisons of samples within each gender or age group (Additional file 6: Table S5). To uncover potentially site-specific correlations [10], correlations were analyzed by skin sites. Palm sites (both left and right) show the greatest positive correlations between community composition dissimilarity of the two domains. Interestingly, forehead comparisons show a modest but significantly negative correlation, such that two samples with greater bacterial community abundance-weighted phylogenetic distance are significantly associated with a lower fungal taxonomic dissimilarity, and vice versa (Additional file 6: Table S5), suggesting that the magnitude and direction of cross-domain community structure correlation is complex, depending on the skin site examined. Correlations between fungal BCD and bacterial weighted UniFrac distances were consistent to that of bacterial BCD, suggesting that taxonomic or phylogenetic properties of the overall bacterial communities have similar effects on shaping cross-domain correlations in this cohort (Additional file 6: Table S5).

SparCC was computed for the bacterial and fungal OTUs to uncover potential cross-domain correlation relationships. Over 200,000 significant and unique SparCC correlations are present in total for the five sites (Additional file 9: Table S6), with forearms (both left and right sites) containing the most number of significant correlations. Most of these correlations belong to bacteria-bacteria relationships, but fungal-fungal and cross-domain relationships are also present. Cross-domain significant SparCC correlations appear to be the most numerous on the forehead, and least numerous at forearm sites (Additional file 10: Table S7). More significant pairings of OTUs from different phyla are involved in co-exclusion correlations, regardless of skin site (Additional file 11: Figure S4). On the other hand, there is increased tendency for taxa of the same genera to present co-abundance than co-exclusion correlations. Conversely, OTUs of same or different domain show no preference for significant co-abundance or exclusion correlations, possibly due to the diversity between OTUs within the broad taxonomic classification (Additional file 11: Figure S4).

**Fig. 4:**
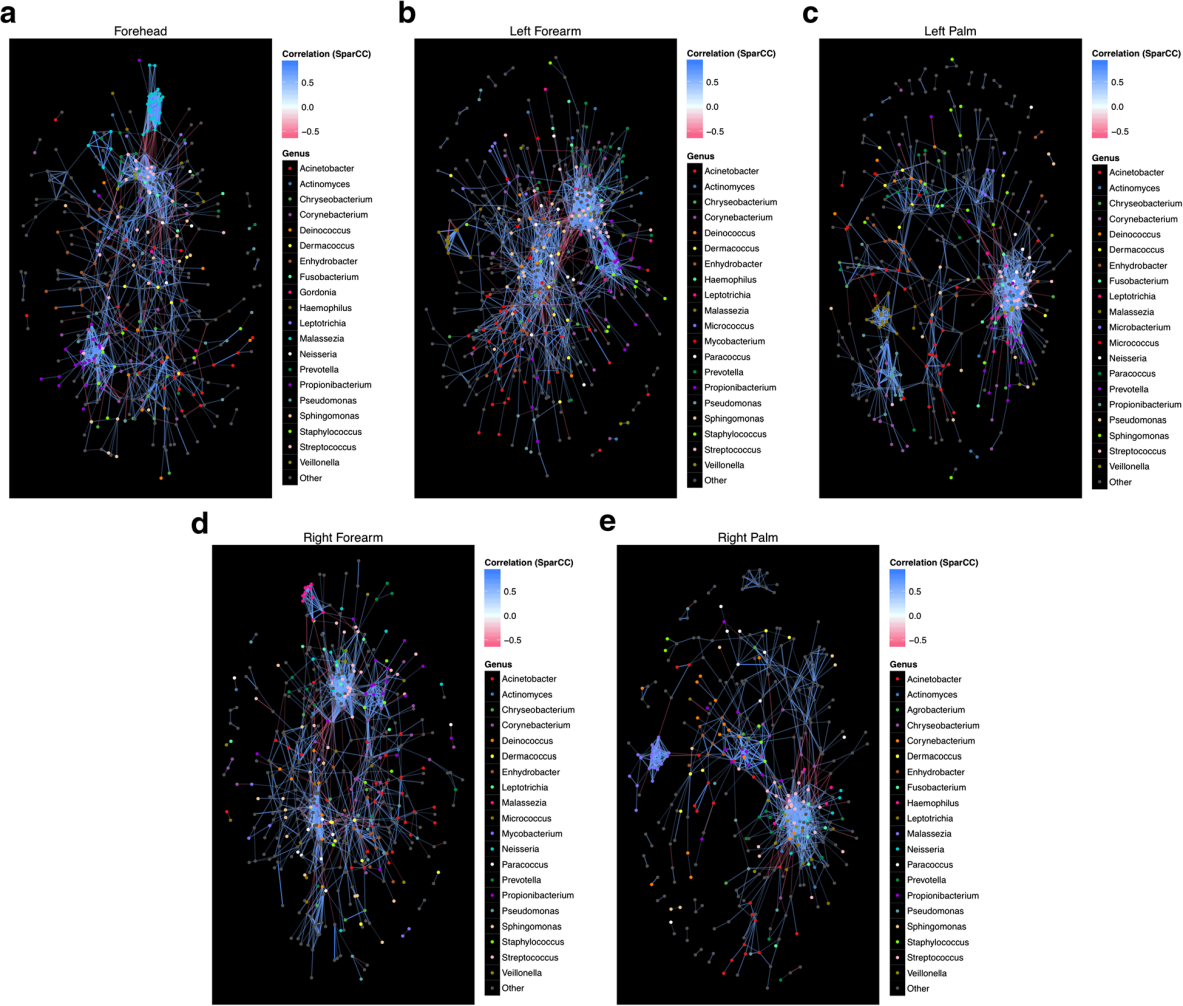
Significant and strong SparCC potential co-abundance and co-exclusion relationships between fungal and bacterial OTUs within skin sites. Separate network plots were constructed for **a** forehead, **b** left forearm, **c** left palm, **d** right forearm, and **e** right palm. Nodes represent OTUs involved in either significant co-abundance (*blue edges*) or co-exclusion (*red edges*) relationships, with the magnitude of the correlation expressed as the intensity of the respective edge colors. The *color* of each node indicates the genus of the OTU. Only significant correlations (two-sided pseudo *p* ≤ 0.05 based on bootstrapping of 100 repetitions) with an absolute correlation magnitude ≥0.5 or ≤−0.5 are presented both for visual clarity and to focus on only the strong correlations. The 20 fungal and bacterial genera most involved in strong and significant correlations are listed, and the remaining genera are grouped as “Other.” All significant relationships (both co-abundance and co-exclusion), sorted by skin site, are provided in Additional file 9: Table S6

Statistically significant and strong correlations (SparCC correlation ≥0.5 or ≤−0.5 and *p* ≤ 0.05) are plotted according to skin site (Fig. 4). Prominent fungal genera appear to be involved in different modes of potential relationships with their fungal and bacterial neighbors. Firstly, regardless of body site, members of *Malassezia* engage in strong potential co-abundance correlations within themselves, which are similar to results seen for bacteria, where intra-genus potential relationships tended to be more positive than negative compared to cross-genus relationships [12]. However, depending on body sites, the OTUs of *Malassezia* also present possible significant co-abundance relationships with *Corynebacterium* (forearm), *Prevotella* (palm), and *Propionibacterium* (forehead). In addition, the fungal colonizer also presents strong co-exclusion correlations with *Pseudomonas* and *Acinetobacter* (both relationships in palms), *Streptococcus* (forehead) and *Enhydrobacter* (forearm), a bacterial genus previously demonstrated to be elevated in Chinese individuals [12, 52].

In another example, OTUs of *Candida* show significant co-abundance relationships with *Pseudomonas* and *Enhydrobacter* and co-exclusion relationships with *Corynebacterium* and *Staphylococcus* on multiple body sites (Additional file 9: Table S6). Interestingly, both co-abundance and co-exclusion correlations between *Candida* and *Enhydrobacter* are observed depending on skin site (co-exclusion on left forearm compared to co-abundance correlations in other sites). Furthermore, two genera are involved in both potential co-abundance and co-exclusion correlations within the same site, as in the case of *Malassezia* and *Propionibacterium* on the forehead, depending on the respective OTUs in question. Furthermore, these fungal OTUs not only present co-abundance and co-exclusion relationships with prominent bacterial OTUs, but also minor and rarer bacterial or fungal OTUs, further stressing the importance of understanding rare OTUs within the microbiome [53].

## Discussion

While cutaneous mycobiome investigations of healthy Asian individuals based in Japan [3, 4, 22, 54–56], Korea [5], and India [57] have been conducted, investigations on Chinese individuals have been limited to prokaryote communities [12, 52, 58]. By selecting only Chinese subjects in our study, our cohort is a representative model of the Chinese population in HK, and while subjects in this study may not represent other Chinese individuals [58], this study is nonetheless fundamental in beginning to understand the skin mycobiome of the Chinese. To date, only a handful of large-scale mycobiome studies of the human body, much fewer dedicated to the analyses of mycobiomes of non-western population groups [59], or mycobiomes’ roles within cross-domain microbial communities [7, 24, 60, 61], have been conducted. Here, we show that common fungal colonizers across western and other Asian populations are abundant and prevalent in this cohort. This study also presents various factors driving skin fungal community variations between sample groups, and site-specific correlational patterns between fungi and co-colonizing bacteria. Moreover, fungi potentially associated with the local environment and lifestyles are also detected in the HK population. We believe that this study sets the stage for understanding the global skin pan-mycobiome. However, as we discuss later, challenges are present in order to systematically assess and compare the fungal communities between global hosts.

Our work here reveals that the conventional UNITE database, compared to the curated database [10], provides less taxonomic coverage, at least in some taxonomic ranks, for skin fungal communities. It must be noted that taxonomic coverage does not equate accuracy. Thus, in our comparison between the databases, the majority of OTUs with taxonomic classification under both databases are classified consistently. While artificially constructed mock communities may help to better ascertain whether the input sequences of known organisms will be correctly identified, as well as the accuracy of both databases, the input sequences will likely be common fungal species that are well represented on both databases, making it challenging to evaluate the ability of the databases in identifying novel sequences. We have shown here that the lack of taxonomic coverage on UNITE also applies to mycobiomes of other populations. Although the coverage of the curated database is enhanced, efforts towards optimizing database coverage are essential. This is exemplified in the inability for both databases to provide genus-level information for over 150 OTUs. Moreover, the selection of reference database should depend on the types of samples and the nature of the investigation, as different fungal databases are available to the scientific community, often designed with different ecological or clinical applications in mind [62–64].

Common epithelial fungi detected globally [10, 25, 61, 65], including *Malassezia*, *Candida*, *Aspergillus*, and *Cryptococcus*, are ubiquitous in HK individuals, suggesting that these fungi constitute a part of the global skin mycobiome core. The average relative abundance of *Malassezia*, the most prominent fungi on our cohort, is within the range of western populations. Species distribution of *Malassezia* shows an overall predominance of *M. restricta*. From this and other studies, different *Malassezia* species predominate depending on sites, individuals, and groups [6, 23, 45, 66, 67]. The current study, based on amplicon sequencing, is limited to taxonomic identification without an understanding of the species- and strain-specific physiologies and pathogenic potentials [3, 22, 54, 68]. Shotgun metagenomics will reveal information regarding metabolic, virulence, and antifungal resistance properties of established and novel species and strains, which may play roles in health risk variations in different hosts as a result of their biogeography patterns [24, 69].

Similar to our previous bacterial community characterization of the same cohort [12], interpersonal fungal community richness, diversity, and compositional differences are observed. However, households appear to affect fungal community membership, but not composition, between occupants, an observation that is in dissonance with bacterial data [12, 15, 70]. Given urbanites spend most of their lives indoors (which has its own indoor mycobiome influenced by the outdoors and shows distance-dependent compositional variation [71, 72]), the skin may act as a collecting ground for fungi within indoor locations. Assuming that fungi on skin come from the indoors by passive exposure and active contact with household surfaces or items, we suggest that the composition variations seen between cohabiting individuals result from individualized fungal exposures, even within a household. In other words, occupant and/or additional factors may personalize the community compositions and structures of different cohabitants. For example, the integrated exposure differences in lifestyles of cohabitants are sufficient to alter their mycobiomes, such that the extents of variations are indistinguishable from that of non-cohabitants. Coincidentally, within sites, only the right palm, the site likely to be exposed to households’ ecosystems via contact, shows a significant household influence on fungal community composition dissimilarity. Given all but one of the individuals in this study are right-handed, one can hypothesize that the relationships between fungal composition of individuals and immediate environments depend on whether passive exposure or active contact is considered. Lack of household effect in community composition may indicate that OTUs shared between cohabitants are low in relative abundances. Residents can pick up individual-specific, low-abundance OTUs through personalized contact and exposure (which explains the membership significance between cohabitants, and the lack thereof composition significance). While we cannot conclusively verify that individualized exposures and contact give rise to the observations, studies combining analyses of human and indoor microbial communities [13, 15, 25, 73–75] can perhaps untangle the complex nature of their relationships. It may be that physical proximity of different people at a local scale may not be inferred by analyzing the similarities of their mycobiomes. Rather, personal exposures and activities may play a crucial part in shaping the skin mycobiome, a hypothesis with implications in microbial forensics [76–78].

Positive cross-domain correlations in community diversity and composition dissimilarities are detected. However, individuality of microbiomes may mean that the nature of relationships within the microbial assemblages (bacterial or fungal) are host- and/or site-specific [10]. Body site may also govern correlations between how dissimilar the bacterial and fungal communities are between any two samples. Consistent with this, we have identified site-specific positive and negative cross-domain correlations at community and OTU levels. While correlation does not equate interaction, coexistence (or polymicrobial encounter [11]) is the first requirement for microbial interaction to occur. Cross-domain interactions take place in various ecosystems, involving physical contact, quorum, and other cell-to-cell metabolite and signaling mechanisms, altering physiochemical properties of their immediate environments [11]. In this study, co-abundance correlations were detected between *Candida* and *Pseudomonas*, genera known to coexist in respiratory tracts of cystic fibrosis patients [60], and the latter has been shown to produce molecules affecting former’s physiology [79, 80]. Nonrandom correlations will potentially facilitate metabolic, ecological, and interactive processes to take place in complex environments [60, 81, 82]. From a clinical perspective, cross-domain relationships have been shown to affect physiology, pathogenicity, and virulence of organisms, as well as their interaction with the host’s immunity [83], and changes in correlation patterns between the two domains have been associated with dermatopathology [61]. While some correlations detected here may be the result of stochastic co-colonizations, some significant correlations observed may be biologically important for microbes and hosts. Also, while site-specific cross-domain correlational patterns at a community level are detected, the variation in statistical effect sizes, especially for comparisons involving samples within individuals, may overestimate the effect of the statistical significance presented. Future multi-“omic” analyses and cultivation assays [84] will undoubtedly provide additional insight into the nature and mechanisms of potential cross-domain interactions that amplicon-sequence and statistical analyses cannot. Furthermore, it will also be of interest to elucidate the dynamics of the mycobiome and its relationships with bacterial community and ascertain whether cross-domain correlational trends over time are also personalized. Previous temporal analyses of skin mycobiome reveal site and individual dependencies on the dynamic and stability of fungal communities [85]. It is currently unknown whether differences in dynamics of mycobiomes occur between global populations. Therefore, future longitudinal analyses of the skin mycobiome in Chinese and other populations will extend our understanding of fungal community dynamics and how mycobiome change within a site correlate with bacterial community variations over time.

Similar to bacteria [12, 17–19], the skin fungal community may exist as a global pan-mycobiome that is greater than the mycobiome of any single individual or population. Consistent with this, fungi that are potentially associated with local environments and lifestyle practices (i.e., geography-specific), including OTUs belonging to *Debaryomyces*, *Cordyceps*, *Auricularia*, and *Stemphylium*, are detected in HK subjects. These genera include fungi associated with mangrove areas immediately adjacent to HK [46], potential pathogens of Chinese garlic [49], and prominent fungi of Chinese cuisines and medicines. While *Debaryomyces* and *Cordyceps* are, respectively, detected in the studies of Adams et al. [25] and Zhang et al. [4], the other “local” genera are not detected in the number of North American and Asian mycobiome studies cited here. Examining the skin pan-microbiome (which also applies to the pan-mycobiome) in detail potentially requires untangling the possible effects of local external environments and lifestyles with physiological and culture-related differences between population groups that may also influence microbial communities [86]. The ability to separate these effects, albeit challenging, will allow scientists to understand how these factors may individually help increase the microbial community on skin across populations. If immediate environments and host activities do shape skin fungal assemblages as demonstrated for bacterial communities [14, 18], future global pan-mycobiome analyses will require a thorough understanding of fungal communities of nearby terrains, as well as the daily activities of host populations. Our observations here do not by themselves provide evidence that these environmental fungi on skin originate from the local environment, and future chamber-based works will allow more controlled assessment of microbial flow between skin and the local environment [35, 87].

The global pan-mycobiome will change our perspective on the breadth of fungal diversity that can inhabit our skin. More importantly, understanding the pan-mycobiome will challenge the notion that mycobiome-directed therapies will have comparable efficacies in global populations. Future multi-cohort comparative investigations of the skin mycobiome should focus on fungi present in specific populations, the potential ecological sources of these fungi, and how these fungi help expand the pan-mycobiome. Unfortunately, effective comparison of mycobiomes between global populations is currently hampered by a lack of standardization or consistency in collecting, processing, and analyzing fungal data. Our study is limited to the characterization of a single non-western cohort, representing an appropriate first step towards unveiling the global pan-mycobiome. Our comparative analyses with the other American studies are suboptimal, because an effective examination of the global community data requires that the studies being compared maintain consistent methodologies in sample collection, DNA extraction, sequencing primers and platforms, and bioinformatics and statistical analyses [18, 19, 58]. Variation from any of these steps will potentially preclude deciphering driving forces behind microbiome variations [38, 88–90]. However, skin mycobiome characterization lags far behind that of bacterial community analyses, and the handful of large-scale skin fungal community data available at present [10, 25, 61, 65] differ in methods adopted, making pan-mycobiome assessment challenging. Therefore, in order to fully determine the size of the global cutaneous fungal community, greater efforts towards method standardization will be of paramount importance, such that studies conducted in different laboratories can be more effectively compared.

## Conclusions

In summary, this study compares the utility of two different fungal reference databases in providing taxonomic information for the skin mycobiome of a Chinese cohort. While major genera prevalent in western populations are also detected here, fungi potentially linked to local environments and life practices are also detected. Furthermore, this study demonstrates the site-specificity of cross-domain correlations at both community and organism levels. Through this study, we set the stage for determining the global skin mycobiome by characterizing a non-western cohort. Such information will pave way for understanding the human pan-mycobiome and uncover relationships between microbes important for ecology, metabolism, health, and disease.

## Additional files

Additional file 1: Table S1. OTU counts and taxonomic information of all samples included in this study. (XLSX 579 kb) Additional file 2: Table S2. Taxonomic coverage of UNITE and curated databases for skin mycobiomes of HK and American cohorts, and accuracy comparison for HK cohort. (XLSX 99 kb) Additional file 3: Table S3. Relative abundances of genera and *Malassezia* species across samples, households, and individuals. (XLSX 80 kb) Additional file 4: Table S4. Fungal α- and β-diversity significances (*p* values) between sample variables, and β-dissimilarities of intra- and intergroup comparisons. (XLSX 37 kb) Additional file 5: Figure S1. Fungal α-diversity differences between age groups, skin sites, and gender. Fungal α-diversity by (a) age group based on observed number of OTUs, as well separated by skin site and grouped by gender using (b) Shannon and (c) Simpson α-diversity indices. Fungal richness data was normalized to a read depth of 1175 reads/sample. (TIF 3389 kb) Additional file 6: Table S5. α-, β-diversities and correlations of fungal and bacterial skin communities. (XLSX 3371 kb) Additional file 7: Figure S2. Density plot of pairwise Bray-Curtis dissimilarity comparison between samples within a skin site. Normalized dissimilarity between two samples within the (a) forehead, (b) left forearm, (c) left palm, (d) right forearm, and (e) right palm, are plotted. Curves are colored according to whether samples being compared come from cohabiting individuals or non-cohabiting individuals. Vertical dashed lines correspond to mean dissimilarities for the comparison groups. (TIF 6955 kb) Additional file 8: Figure S3. Density plot of pairwise binary Jaccard distance comparison between samples within a skin site. Normalized dissimilarity between two samples within the (a) forehead, (b) left forearm, (c) left palm, (d) right forearm, and (e) right palm, are plotted. Curves are colored according to whether samples being compared come from cohabiting individuals or non-cohabiting individuals. Vertical dashed lines correspond to mean distances for the comparison groups. (TIF 6945 kb) Additional file 9: Table S6. Significant SparCC correlations between OTUs of fungal and bacterial origins divided by body sites. (XLSX 32215 kb) Additional file 10: Table S7. Proportions in percentages of significant SparCC correlations based on domain relationships between OTUs. (DOCX 61 kb) Additional file 11: Figure S4. Density plots of cross-domain/phylum/genus SparCC correlations across skin sites. Density plots of significant correlations (based on pseudo *p* values ≤0.05 following bootstrapping of 100 repetitions) between OTUs of same (i.e., intra, blue curve) or different (i.e., inter, red curve) domain/phylum/genus for each of (a–c) forehead, (d–f) left forearm, (g–i) left palm, (j–l) right forearm, and (m–o) right palm sites. The plots are broken down into taxonomic level of comparison: (a, d, g, j, m) cross-domain, (b, e, h, k, n) cross-phylum, and (c, f, i, l, o) cross-genus. OTUs with unclassified or unknown taxonomic assignments were removed from this analysis. (TIF 3087 kb)

## Abbreviations

ANOSIM: Analysis of similarities
BCD: Bray-Curtis distance
BE: Built environment
BLAST: Basic Local Alignment Search Tool
HK: Hong Kong
JD: Jaccard distance
KW: Kruskal-Wallis
NCBI: National Center for Biotechnology Information
OK: Oklahoma
OTU: Operational taxonomic unit
PCR: Polymerase chain reaction
QIIME: Quantitative Insights Into Microbial Ecology
RNA: Ribonucleic acid
SparCC: Sparse Correlation for Compositional Data
UNITE: User-friendly Nordic ITS Ectomycorrhiza Database
USA: United States
