# Supplementary figures and images for: Skin fungal community and its correlation with bacterial community of urban Chinese individuals
Source: Microbiome. 2016 Aug 24;4(1):46. doi: 10.1186/s40168-016-0192-z (PMC4997687; doi:10.1186/s40168-016-0192-z)

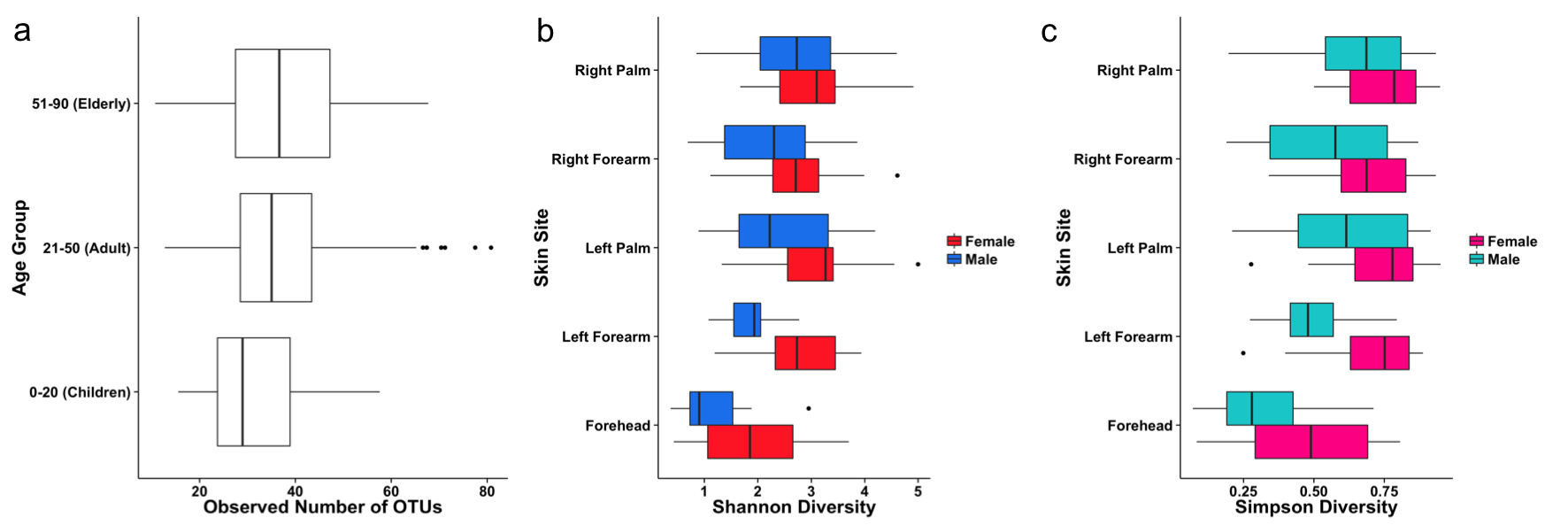

Supplement: Additional file 5: Figure S1. — Fungal α-diversity differences between age groups, skin sites, and gender. Fungal α-diversity by (a) age group based on observed number of OTUs, as well separated by skin site and grouped by gender using (b) Shannon and (c) Simpson α-diversity indices. Fungal richness data was normalized to a read depth of 1175 reads/sample. (TIF 3389 kb) [file 40168_2016_192_MOESM5_ESM.tif]

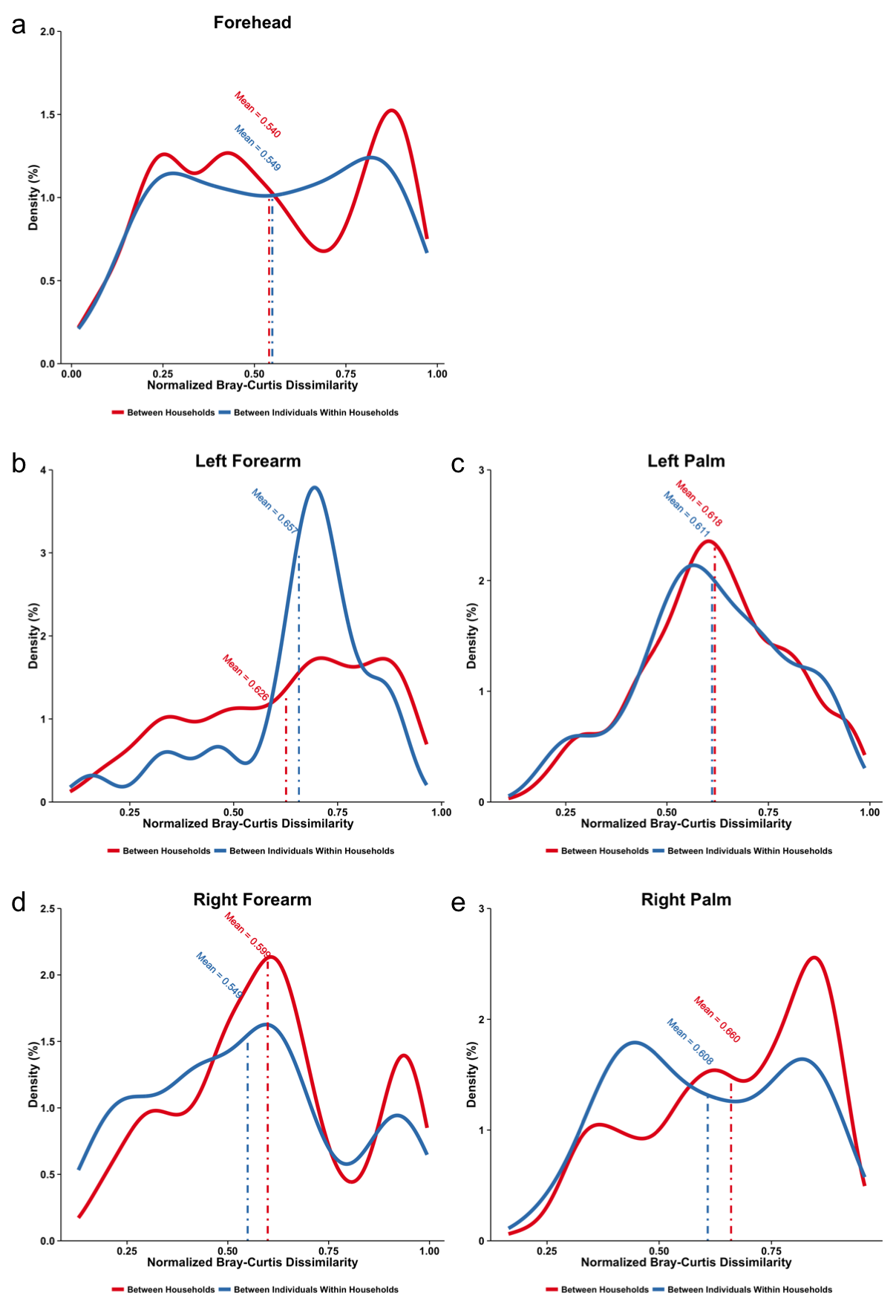

Supplement: Additional file 7: Figure S2. — Density plot of pairwise Bray-Curtis dissimilarity comparison between samples within a skin site. Normalized dissimilarity between two samples within the (a) forehead, (b) left forearm, (c) left palm, (d) right forearm, and (e) right palm, are plotted. Curves are colored according to whether samples being compared come from cohabiting individuals or non-cohabiting individuals. Vertical dashed lines correspond to mean dissimilarities for the comparison groups. (TIF 6955 kb) [file 40168_2016_192_MOESM7_ESM.tif]

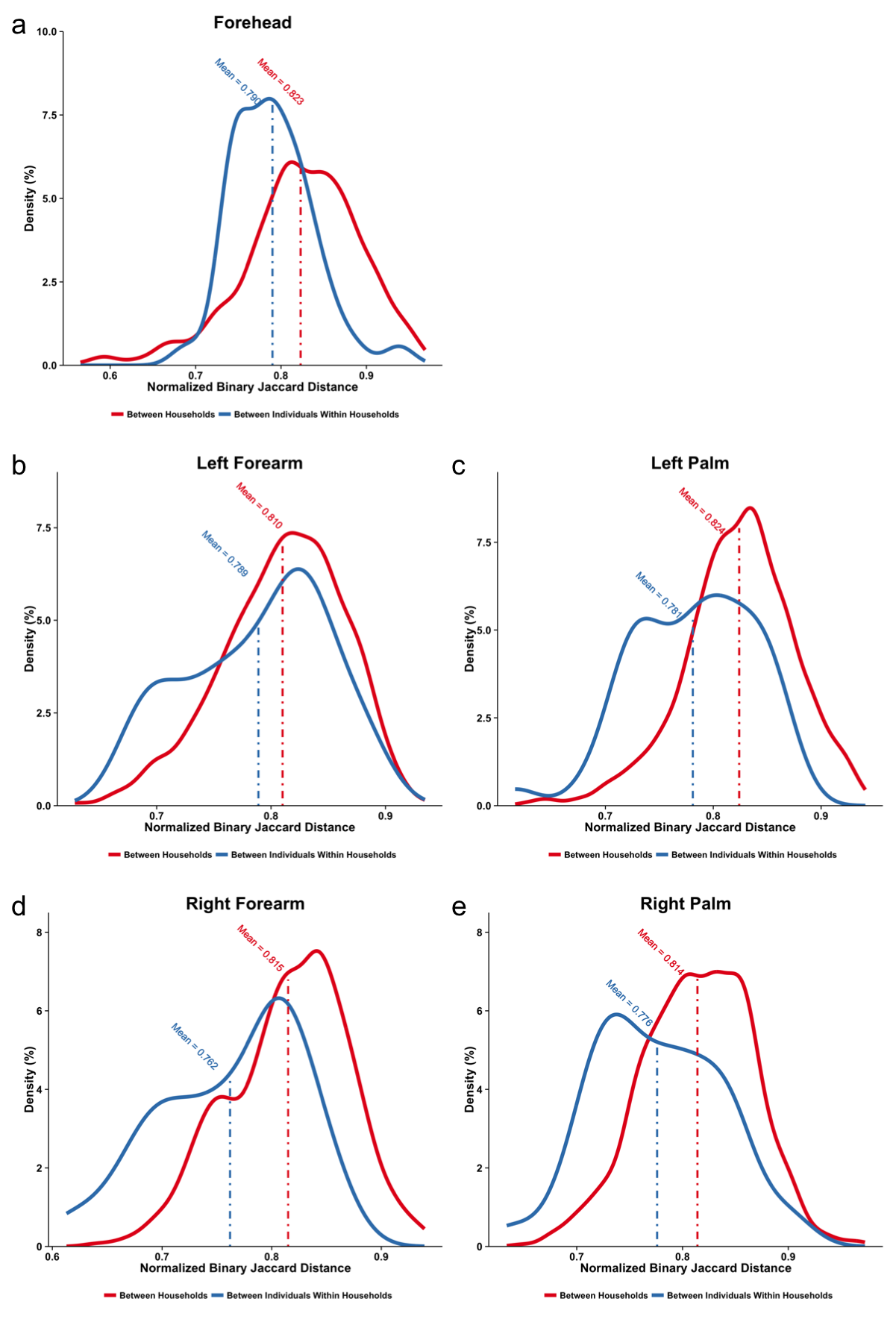

Supplement: Additional file 8: Figure S3. — Density plot of pairwise binary Jaccard distance comparison between samples within a skin site. Normalized dissimilarity between two samples within the (a) forehead, (b) left forearm, (c) left palm, (d) right forearm, and (e) right palm, are plotted. Curves are colored according to whether samples being compared come from cohabiting individuals or non-cohabiting individuals. Vertical dashed lines correspond to mean distances for the comparison groups. (TIF 6945 kb) [file 40168_2016_192_MOESM8_ESM.tif]

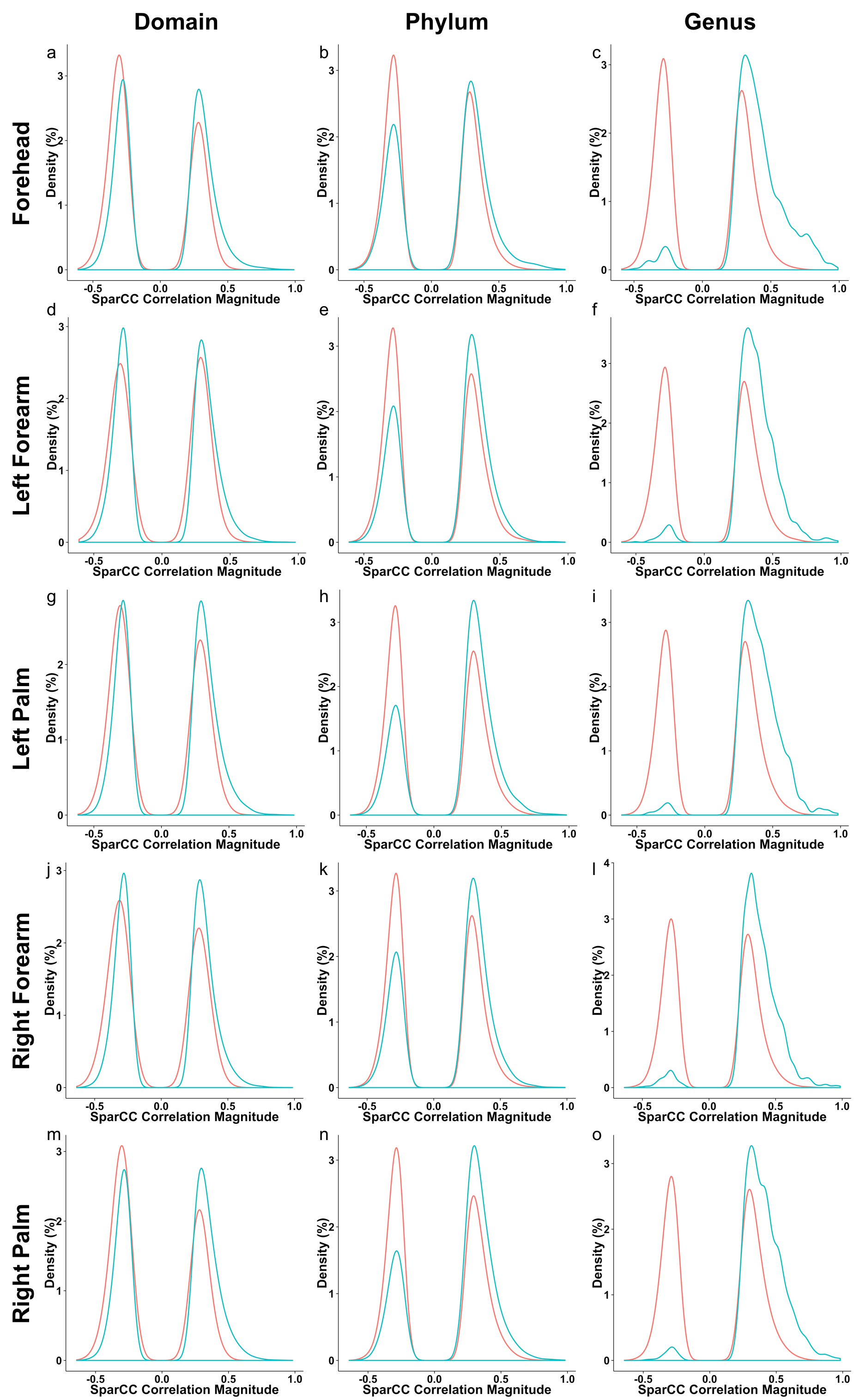

Supplement: Additional file 11: Figure S4. — Density plots of cross-domain/phylum/genus SparCC correlations across skin sites. Density plots of significant correlations (based on pseudo p values ≤0.05 following bootstrapping of 100 repetitions) between OTUs of same (i.e., intra, blue curve) or different (i.e., inter, red curve) domain/phylum/genus for each of (a–c) forehead, (d–f) left forearm, (g–i) left palm, (j–l) right forearm, and (m–o) right palm sites. The plots are broken down into taxonomic level of comparison: (a, d, g, j, m) cross-domain, (b, e, h, k, n) cross-phylum, and (c, f, i, l, o) cross-genus. OTUs with unclassified or unknown taxonomic assignments were removed from this analysis. (TIF 3087 kb) [file 40168_2016_192_MOESM11_ESM.tif]
