# Supplementary material for: Skin fungal community and its correlation with bacterial community of urban Chinese individuals
Source: Microbiome. 2016 Aug 24;4(1):46. doi: 10.1186/s40168-016-0192-z (PMC4997687; doi:10.1186/s40168-016-0192-z)
Supplement: Additional file 10: Table S7. — Proportions in percentages of significant SparCC correlations based on domain relationships between OTUs. (DOCX 61 kb) [file 40168_2016_192_MOESM10_ESM.docx]

**Additional File 10: Table S7. Proportions in percentages of significant SparCC correlations based on domain relationships between OTUs.**

| **Skin site** | **No. of unique correlations^a^** | **Bacteria/Bacteria (%)** | **Fungi/Bacteria (%)** | **Fungi/Fungi (%)** |
| --- | --- | --- | --- | --- |
| **Forehead** | 42,681 | 90.3 | 8.6 | 1.1 |
| **Left forearm** | 56,944 | 97.5 | 2.4 | < 0.2 |
| **Left palm** | 35,243 | 94.2 | 5.2 | < 0.6 |
| **Right forearm** | 43,265 | 96.7 | 3.1 | < 0.3 |
| **Right palm** | 28,631 | 96.1 | 2.9 | 1.0 |

^a^ Significant SparCC correlations are based on two-sided pseudo *p-*values following bootstrapping support of 100 repetitions. Significant correlations where one or both OTUs were of unidentified domains were not included. Number of unique correlations shown here are discrepant from that of Additional File 9: Table S6. Significant correlations between two OTUs in Additional File 9: Table S6 are listed as two separate correlations (OTU1 with OTU2 as one correlation, and OTU2 and OTU1 listed as a separate correlation, therefore not unique). Percentages of unique correlations regarded as bacterial-bacterial, bacterial-fungal, or fungal-fungal are listed for each skin site.
